# Mucin glycans drive oral microbial community composition and function

**DOI:** 10.1038/s41522-023-00378-4

**Published:** 2023-03-23

**Authors:** Chloe M. Wu, Kelsey M. Wheeler, Gerardo Cárcamo-Oyarce, Kazuhiro Aoki, Abigail McShane, Sujit S. Datta, Jessica L. Mark Welch, Michael Tiemeyer, Ann L. Griffen, Katharina Ribbeck

**Affiliations:** 1 Department of Biological Engineering, Massachusetts Institute of Technology, Cambridge, MA USA; 2 Microbiology Graduate Program, Massachusetts Institute of Technology, Cambridge, MA USA; 3 Complex Carbohydrate Research Center, University of Georgia, Athens, GA USA; 4 Department of Cell Biology, Neurobiology and Anatomy, Medical College of Wisconsin, Milwaukee, WI USA; 5 Chemical and Biological Engineering, Princeton University, Princeton, NJ USA; 6 Department of Microbiology, The Forsyth Institute, Cambridge, MA USA; 7 Department of Dentistry, Nationwide Children’s Hospital, Columbus, OH USA; 8 Divisions of Biosciences and Pediatric Dentistry, College of Dentistry, The Ohio State University, Columbus, OH USA

**Keywords:** Microbial ecology, Microbiome, Biofilms, Bacteria

## Abstract

Human microbiome composition is closely tied to health, but how the host manages its microbial inhabitants remains unclear. One important, but understudied, factor is the natural host environment: mucus, which contains gel-forming glycoproteins (mucins) that display hundreds of glycan structures with potential regulatory function. Leveraging a tractable culture-based system to study how mucins influence oral microbial communities, we found that mucin glycans enable the coexistence of diverse microbes, while resisting disease-associated compositional shifts. Mucins from tissues with unique glycosylation differentially tuned microbial composition, as did isolated mucin glycan libraries, uncovering the importance of specific glycan patterns in microbiome modulation. We found that mucins shape microbial communities in several ways: serving as nutrients to support metabolic diversity, organizing spatial structure through reduced aggregation, and possibly limiting antagonism between competing taxa. Overall, this work identifies mucin glycans as a natural host mechanism and potential therapeutic intervention to maintain healthy microbial communities.

## Introduction

The human body harbors trillions of diverse microbes in complex communities across our mucosal surfaces, which exhibit remarkable stability over time. Maintaining microbial homeostasis is essential to human health, but host mechanisms to select for beneficial members and establish coexistence between competing microbes are poorly understood. Our microbiotas predominantly reside in mucus, a complex viscoelastic matrix that coats all non-keratinized epithelial surfaces in the body. Disruptions in mucus including altered viscoelasticity and glycosylation are associated with numerous pathologies^1–4^ and microbiome imbalance (dysbiosis)^2,5–7^, highlighting the importance of an intact mucus barrier for health.

**Fig. 1:**
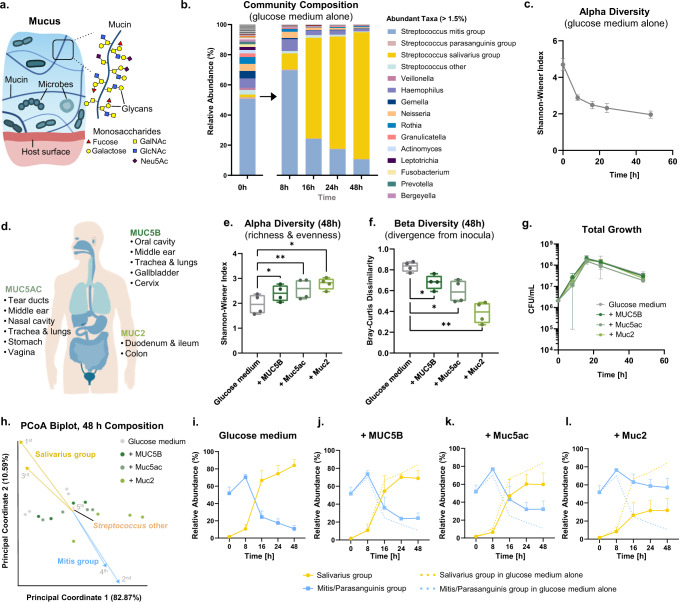
Mucins from different mucosal niches promote diverse oral microbial communities. **a** Schematic of microbial communities embedded in networks of mucin glycoproteins. GalNAc: N-acetyl galactosamine; GlcNAc: N-acetyl glucosamine; Neu5ac: sialic acid. **b** Relative abundance from 16S rRNA sequencing of inoculating communities derived from pooled human saliva. Each stacked bar represents the average relative abundances including replicates from two inoculating communities (*n* = 4). **c** Taxonomic diversity (Shannon-Wiener Index) over time in glucose medium without mucins. Error bars indicate the standard error of the mean (s.e.m.) (*n* = 4). **d** Primary gel-forming mucin types isolated from human saliva (MUC5B), porcine gastric mucus (Muc5ac) and porcine intestinal mucus (Muc2) representative of mucosal niches across the human body. **e**, **f** Alpha (**e**) and beta (**f**) diversity of microbial communities cultured in medium with or without mucins (48 h, *n* = 4). In (**e**, **f**), center lines indicate the medians, box limits indicate upper and lower quartiles, and whiskers indicate minimum and maximum values. Each point represents an independent replicate. Significant differences relative to medium without mucin were assessed using repeated-measures one-way analysis of variance (ANOVA) with Dunnett’s multiple comparison test. *p < 0.05; **p < 0.01. **g** Total growth measured by counting colony-forming units (CFUs) in glucose medium with or without mucins. Each point represents the average CFU/mL (*n* = 3), and error bars indicate the standard deviation (s.d.). **h** PCoA biplot of 16 S rRNA sequencing data showing community separation by culture environment (48 h, *n* = 4). Each point represents an independent replicate. Arrow magnitude reflects influence on overall discrimination, and the 5 most contributing individual taxa are represented. **i–l** Relative abundance of *Streptococcus* groups in glucose medium without (**i**) or with (**j**) MUC5B, (**k**) Muc5ac, or (**l**) Muc2. Each point represents the average relative abundance, and bars represent s.e.m. (*n* = 4). All experiments were performed using inoculating communities 1 and 2, as depicted in Supplementary Fig. 1. The schematic in (**a**) contains components (mucin polymers, bacteria) that are adapted with permission^15^, originally published in *The FEBS Journal*.

Many structural and biological functions of mucus center around mucins, large glycoproteins comprised of a peptide backbone densely coated with distinct branching sugar chains (glycans) (Fig. 1a). Mucins can interact with microbes in various ways. For instance, bacteria that encode machinery to degrade complex carbohydrates can utilize whole mucus^8,9^ or purified mucin^10,11^ as a nutrient source. Mucin can also bind to microbes^12–14^ and mediate their spatial organization^15^. Moreover, mucins and their chemically-diverse *O*-linked glycans can influence microbial behaviors including biofilm formation^16–18^, communication^16^, and competition^18,19^. These numerous functions suggest a critical role for mucins in maintaining microbial homeostasis, but mucins’ impact in the context of complex microbial communities is largely understudied. Recently, mice fed a diet supplemented with mucin glycans were shown to exhibit differences in microbial composition^20^, opening new questions on the importance of mucin glycan biochemistry, and the underlying mechanisms of interaction with microbes.

Here, we investigate how mucins purified from multiple mucosal niches including human saliva, porcine gastric mucus, and porcine intestinal mucus (which vary in glycosylation), and isolated mucin glycan libraries ranging in composition and structural complexity, each specifically affect microbial communities. We focused on the oral cavity, which contains a diverse salivary microbiota^21^ where compositional shifts are linked to various pathologies^22,23^. The accessibility of the oral niche enabled us to capture native host–microbe interactions, as both mucins and microbial communities could be isolated from human saliva. Additionally, while natural oral communities exhibit broad metabolic potential^24^ and striking spatial distribution patterns^25,26^, the impact of mucin on these phenotypes has not yet been determined.

Testing how host factors influence bacterial communities is challenging in vivo due to individual variation and lack of experimental control. Meanwhile, in vitro setups often fail to capture native host functions and community complexity. To bridge this gap, we used a tightly controlled culture-based system to dissect how mucins regulate oral microbial communities. We identified that mucins isolated from different mucosal tissues share a conserved function in promoting coexistence between diverse community members and resisting dysbiotic shifts associated with disease, while distinct sets of glycan structures determine the specific microbial composition. Beyond composition, we found evidence that mucins expand the landscape of metabolic activity and shape more spatially dispersed community structures. These results suggest mucin glycans as a powerful natural host tool for establishing healthy microbiotas.

## Results

### Oral microbial communities cultured with mucins have increased taxonomic diversity

To assess how mucins shape microbiota composition, we inoculated native oral communities derived from human saliva into a glucose-based, chemically-defined medium developed to cultivate oral bacteria^27^, in the absence or presence of mucins. Different medium formulations likely influence composition; this experimental setup tests mucins’ effects in the context of potential selective enrichment driven by a glucose-based medium. We then profiled their composition over time using 16S ribosomal RNA (rRNA) gene sequencing. These starting inocula were taxonomically rich, containing at least 54 genera across eight phyla (Fig. 1b, Supplementary Fig. 1).

When grown in glucose-based medium alone, the communities underwent a rapid loss of diversity (Fig. 1b, c). To evaluate mucin’s impact on community composition, we supplemented the glucose-based medium with mucins from human saliva (primarily MUC5B), porcine gastric mucus (primarily Muc5ac, the counterpart to human MUC5AC), and porcine intestinal mucus (primarily Muc2, the counterpart to human MUC2) (Fig. 1d). Importantly, mucins were purified from native tissues to retain their key viscoelastic properties^28,29^ and dense coatings of complex glycans^29^ which can be lost in commercial preparations. Each mucin partially mitigated the collapse in microbial diversity (Fig. 1e), and shaped communities that more closely resembled the composition of the starting inocula than those grown without mucin (Fig. 1f). The overall growth and total extracted DNA quantity remained similar across the environments (Fig. 1g, Supplementary Fig. 2). Thus, the observed compositional patterns are not conflated with drastic changes in total biomass or sequenced DNA content. These findings reveal a conserved high-level function across mucin types to maintain more diverse communities closer in composition to native microbiota.

### Mucins favor potentially beneficial bacteria and resist dysbiosis

To identify taxa that differentiate communities grown in the absence or presence of MUC5B, Muc5ac, or Muc2, we performed principal coordinates analysis (PCoA) on the sequencing data. The community composition exhibited clear patterning according to culture environment (Fig. 1h), and the first two principal coordinates captured the majority of sample variance (93.46% combined). Of the most influential individual taxa, the first and third taxa belong to the Salivarius group streptococci (including *Streptococcus salivarius, Streptococcus vestibularis*, and *Streptococcus thermophilus*), while the second and fourth taxa belong to the Mitis group streptococci (including *Streptococcus mitis, Streptococcus oralis*, and *Streptococcus infantis*) (Fig. 1h).

In the glucose-based medium alone, we observed a loss of health-associated Mitis and Parasanguinis streptococci over time and concomitant outgrowth of Salivarius streptococci (Fig. 1i). When mucin was added to the glucose medium, Mitis and Parasanguinis streptococci were retained while Salivarius streptococci outgrowth was limited (Fig. 1j−l). Lower diversity and shifts in the ratio of oral Mitis to Salivarius streptococci are correlated with disease, including dental caries^30^ and Crohn’s disease^31^, suggesting a protective role of mucins against microbial dysbiosis. Further, hierarchical clustering analysis of the sequencing data revealed that communities clustered based on the particular mucin type they were grown with (Supplementary Fig. 3), indicating that microbial composition may depend on the specific polymer structure and biochemistry of the mucin type secreted from a given body surface.

### Mucin glycans impact community diversity and membership depending on specific structural patterns

**Fig. 2:**
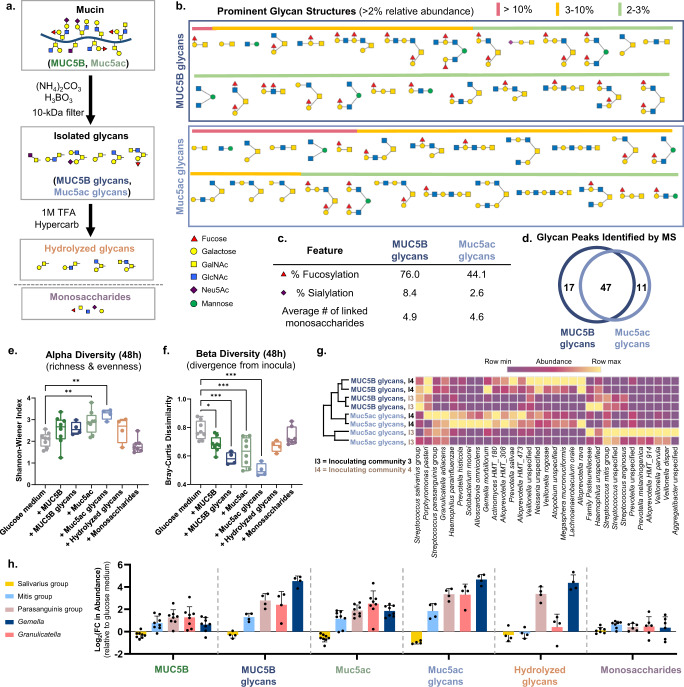
Isolated mucin glycans promote microbial diversity and support specific microbial taxa in a structure-dependent way. **a** Summary of the different mucins and glycan pools compared in this experiment. **b** Prominent glycan structures identified by MS analysis. Bars above each structure are colored by relative abundance. **c** Fucosylation and sialylation levels and average number of linked monosaccharides of MUC5B and Muc5ac glycans. **d** Overlap between libraries of glycans isolated from MUC5B and Muc5ac. **e**, **f** Alpha (**e**) and beta (**f**) diversity of microbial communities cultured in medium with or without mucin glycans for 48 h. In (**e**, **f**), center lines indicate the medians, box limits indicate upper and lower quartiles, and whiskers indicate minimum and maximum values. Each point represents an independent replicate. Significant differences relative to medium alone were assessed using repeated-measures one-way ANOVA with Dunnett’s multiple comparison test. *p < 0.05; **p < 0.01; ***p < 0.001. **g** Hierarchical clustering analysis of communities grown with MUC5B glycans or Muc5ac glycans. The dendrogram represents average linkage clustering (1-Pearson distance metric) of communities based on microbial composition (48 h). The heatmap shows the relative abundance of microbial taxa >0.1% abundance (48 h). Each column represents and independent replicates. **h** Effect of mucins and mucin glycans on the lower relative abundance of select taxa. Bar length represents the mean abundance change relative to medium alone. Each point represents the change for an independent replicate, and error bars indicate the s.e.m. All experiments were performed with inoculating communities 3 and 4 (see also Supplementary Fig. 1). Analysis for medium alone, MUC5B, Muc5ac, and monosaccharides includes duplicates for each inoculating community 1–4 (*n* = 8). Analysis for MUC5B glycans, Muc5ac glycans, and hydrolyzed glycans includes inoculating communities 3 and 4 (*n* = 4). In (**e**, **f**), data for medium alone, MUC5B, and Muc5ac with inoculating communities 1 and 2 are duplicated from Fig. 1e, f for comparison.

To directly test whether mucin glycosylation influences community composition, we created pools of isolated glycans from MUC5B and Muc5ac using non-reductive alkaline β-elimination, which preserves *O*-glycans in their native form (Fig. 2a). Mass spectrometric analysis identified over 50 glycan peaks in each library. Unique and shared structures were detected as prominent components of each mucin type; glycan lengths, branching patterns, and terminal modifications defined library diversities (Fig. 2b–d). Communities grown in mucins and mucin glycans broadly exhibited similar trends in diversity and membership (Fig. 2e, f, h), revealing that glycan structures contribute to mucins’ conserved ability to shape microbial composition. As with the full mucin polymers, glycans impacted composition without significantly altering total growth or extracted DNA content (Supplementary Fig. 4). MUC5B glycans and Muc5ac glycans shaped communities that were more similar to the starting inocula (Fig. 2f), indicating that mucin glycans (not the peptide backbone) are key to maintaining complex community composition. To more closely examine the effects of glycan pools from different tissues, we applied hierarchical clustering to the sequencing data and found that communities clustered primarily based on whether their environment contained Muc5ac glycans or MUC5B glycans, while subclusters formed according to the inoculating community (Fig. 2g). Thus, the precise structural patterns in each mucin glycan pool drive microbial community composition.

To assess the role of glycan structural complexity, we subjected Muc5ac glycans to partial acid hydrolysis, creating a glycan library of reduced complexity^32^, and tested a mixture of the monosaccharide building blocks that comprise mucin glycans (Fig. 2a). The hydrolyzed glycan library had an intermediate effect on diversity relative to mucin glycans and medium alone (Fig. 2e, f), which intriguingly, was not due to partial function across all bacterial groups, but rather changes in select taxa to an extent comparable to those of fully complex Muc5ac and MUC5B glycan pools. Specifically, relative to medium alone, hydrolyzed glycans increased the abundance of Parasanguinis streptococci and *Gemella*, but not Mitis streptococci or *Granulicatella* (Fig. 2h), suggesting that unmodified core structures may be sufficient to enrich certain bacteria, while the presence of fucose, sialic acid, and extended N-acetyl lactosamine chains may be important for maintaining others. As monosaccharides alone were unable to limit Salivarius streptococci or retain these predominant commensals (Fig. 2h), the specific chemical linkages and stereochemistry of mucin glycans may be key to this function. Collectively, these findings reveal that mucin glycans maintain the diversity and composition of microbial communities. The potency and specificity of these effects vary for glycan pools with differing structures and complexities (as the glycans range in the type and extent of branching and the number of monosaccharides per glycan).

### Mucin glycans serve as a nutritive substrate to retain native genera and high diversity

**Fig. 3:**
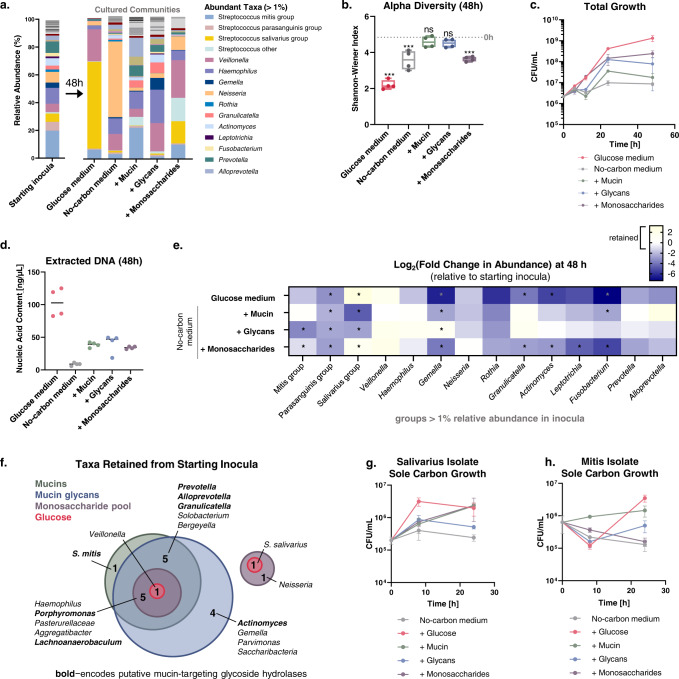
Mucins and mucin glycans as the sole carbon source shape oral communities with native genera and high diversity. **a** Relative abundance of microbial communities cultured with mucins or mucin glycans. Bars show average relative abundances of four replicates including two inoculating communities (*n* = 4). **b** Alpha diversity of microbial communities cultured for 48 h in glucose-based medium, or no-carbon medium with mucins, mucin glycans, or monosaccharides as the sole carbon source (*n* = 4). Center lines indicates the medians, box limits indicate the upper and lower quartiles, and the whiskers indicate minimum and maximum values. Each point represents an independent replicate. Significant differences relative to the native microbiota were assessed by repeated-measures one-way ANOVA with Dunnett’s multiple comparison test. *p < 0.05; **p < 0.01; ***p < 0.001. **c** Total community growth on different carbon substrates over 48 h, where each point represents the average CFU/mL (*n* = 3) and error bars indicate the s.d. **d** Extracted DNA content from samples grown in each carbon environment (*n* = 4). **e** Change in abundance of dominant genera following growth in medium with mucin glycans as the sole carbon source (48 h). The heatmap shows changes in relative abundance compared with starting inocula. Significant differences in taxa abundance were identified by one-way, two-sided *t*-tests. The significance threshold was adjusted by Bonferroni’s correction for multiple comparisons. *p < 0.0036. **f** Venn diagram of genera and streptococci groups retained (log2 fold change [FC] > −1) in cultures with different carbon sources. Bolded genera likely encode putative mucin glycan degradation machinery according to the CAZy database (see also Supplementary Fig. 6). **g**, **h** Growth of Salivarius (**g**) and Mitis (**h**) streptococci strains isolated from native oral communities with glucose, mucin, mucin glycans, or monosaccharides as the sole carbon source. Each point represents the average CFU/mL (*n* = 3), and error bars indicate the s.d. Experiments in (**a–f**) were performed using inoculating communities 3 and 4, as depicted in Supplementary Fig. 1.

To probe the mechanisms by which mucins modulate community composition, we first tested one possibility: that mucin glycans serve as a nutrient source to support specific bacteria. Here, we monitored the composition of oral communities cultured in medium containing mucin, isolated mucin glycans, or a pool of monosaccharides as the sole carbon source. Mucin and mucin glycans supported the growth of taxonomically rich communities, maintaining diversity levels comparable to that of the starting inocula (Fig. 3a, b). Consistent with this finding, hierarchical clustering revealed that inoculating communities clustered most closely with communities cultured in medium with mucin, followed by mucin glycans (Supplementary Fig. 5). We observed differences in growth on each sole carbon source (Fig. 3c, d), but substantial growth on each mucin-based substrate showed that the diverse taxa abundant at 48 h were actively supported within an expanding population (not simply carried over from the starting inocula). Together, these data suggest that mucin glycans facilitate growth of highly diverse communities, and that the structural complexity of these glycans is key to their function.

We next sought to identify community members that benefit from the presence of mucin glycans. Strikingly, mucin glycans retained the majority of dominant genera from the native community over 48 h, whereas the glucose- and monosaccharide-based media exhibited a widespread loss in taxa (Fig. 3e, f). Overall, mucin glycans retained the most groups (15 genera) from the starting community, with mucin supporting most of these groups (11/15 genera) and additionally *S. mitis* (Fig. 3f). Many of these retained groups include strains that encode putative mucin glycan degradation machinery (Fig. 3f, Supplementary Fig. 6). These findings show that mucin glycans can serve as a nutrient source to benefit a wide range of bacteria from native saliva that is lost in glucose- or monosaccharide-based medium.

To further probe the role of mucin-supported nutrition, we isolated strains of Mitis and Salivarius streptococci from native oral communities and characterized their growth when provided glucose, mucin, glycans, or pooled monosaccharides as the sole carbon source. Salivarius exhibited more rapid growth in the glucose medium compared with any of the mucin substrates, whereas Mitis growth was highest in the mucin medium at 8 h (Fig. 3g, h). Individual representative strains for these groups may not represent the heterogeneity of metabolic profiles among oral streptococci and this limitation should be considered in interpreting these results; however, these experiments indicate that nutrition may play a role in the competitive dynamics between Mitis and Salivarius streptococci in the sole carbon source context.

### Mucin glycans support metabolically rich microbial communities

**Fig. 4:**
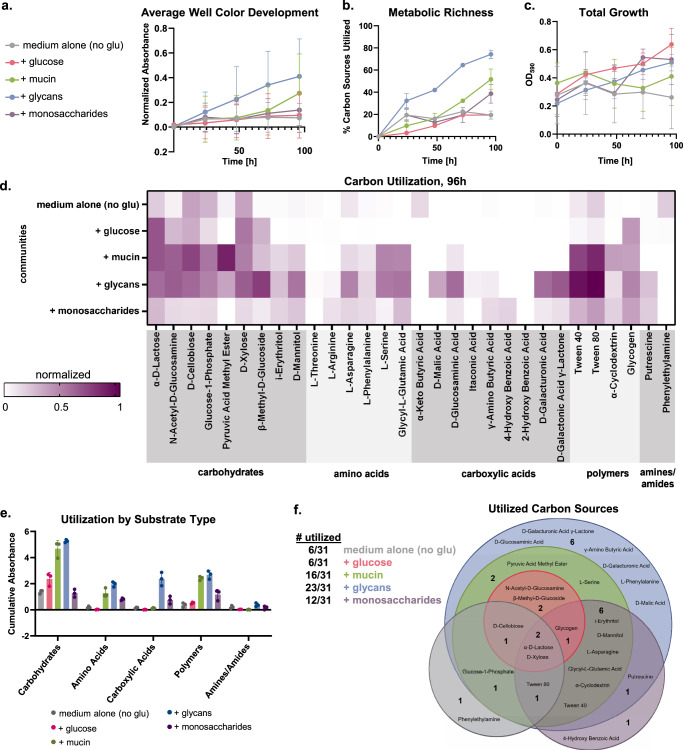
Oral communities grown with mucin glycans utilize diverse metabolic substrates. **a** Average well color development of microbial communities that grew for 24 h in each medium environment, and then were diluted and inoculated into Biolog EcoPlates. Each point represents the average of three replicate, and error bars indicate s.d. **b** Metabolic richness, indicated by % carbon sources utilized (normalized absorbance >0.15). Each point represents the average of three replicates and error bars indicate s.d. **c** Total growth of communities from each environment, as monitored on a separate 96-well plate by OD_590_. Each point represents the average of three replicates and error bars indicate s.d. **d** Heatmap showing normalized absorbance values of carbon utilization for each microbial community at 96 h. Each sample represents the average of three replicates. Carbon utilization profiles from 0 to 72 h are shown in Supplementary Fig. 7. **e** Utilization by substrate type based on 96 h measurements. Each point represents an independent replicate and error bars indicate s.d. (*n* = 3). **f** Venn diagram of carbon substrates utilized by communities from each culture environment at 96 h.

To test whether the taxonomic diversity supported by mucin glycans is linked to more diverse metabolic function, we used Biolog EcoPlates to profile microbial communities’ ability to oxidize various carbon sources. Oral communities were grown for 24 h in chemically-defined medium (including amino acids, vitamins, and other carbon sources) supplemented with either glucose, mucins, mucin glycans, or pooled monosaccharides. These communities were then diluted and inoculated into EcoPlates for metabolic profiling. Communities that had grown in mucin glycans exhibited the highest overall metabolic activity (average well color development) and metabolic richness (fraction of carbon sources utilized), followed by mucins, monosaccharides, then glucose and medium alone (Fig. 4a, b). Overall growth, monitored by OD_590_ readings on a separate 96-well plate, was comparable between the different media (Fig. 4c). More specifically, communities that had grown in mucin glycans were able to utilize a wide range of carbon sources, including carbohydrates, amino acids, and carboxylic acids, while those grown with intact mucin polymers or pooled monosaccharides each utilized a subset of these carbon sources (Fig. 4d–f, Supplementary Fig. 7). Together with the sole carbon source experiments, these findings indicate that mucin glycans shape diverse microbial communities with rich metabolic function, potentially by serving as complex nutritive substrates to enable niche partitioning^33^ and/or by regulating metabolic pathways^34^.

### Mucins spatially organize bacterial communities by reducing surface attachment and aggregation

**Fig. 5:**
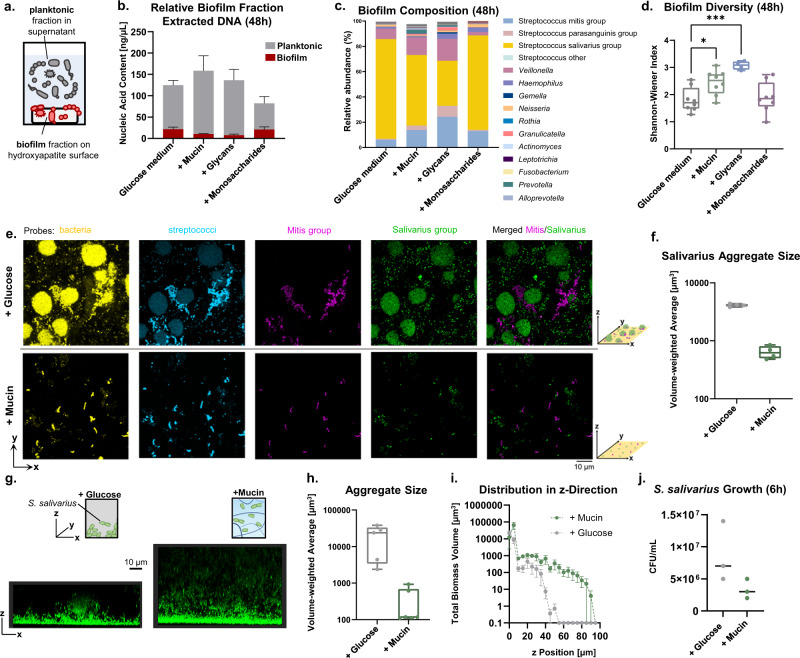
Mucins spatially organize oral microbial communities, reducing surface attachment and aggregation. **a** Schematic of planktonic and biofilm community fractions, grown in a 96-well plate containing hydroxyapatite discs. **b** Relative biofilm fraction of communities grown in mucin and mucin glycans versus medium alone. Bars represent mean ± s.e.m. of four replicates. **c** Relative abundance of biofilm fractions determined from 16S rRNA sequencing. Data represent the average of four replicates. **d** Alpha diversity of microbial communities cultured in medium with mucins or glycans versus medium alone (48 h, *n* = 4). Center lines indicate the medians, box limits indicate upper and lower quartiles, and whiskers indicate minimum and maximum values. Each point represents an independent replicate. Significant differences relative to medium alone were assessed using repeated-measures one-way ANOVA with Dunnett’s multiple comparison test. *p < 0.05; ***p < 0.001. Experiments in (**b–d**) used inoculating communities 3 and 4, as shown in Supplementary Fig. 1. **e** FISH images showing large Salivarius group aggregates (green channel) in glucose-based medium (top row), but not in mucin-based medium (bottom row). Images of individual channels and merged magenta (Mitis) and green (Salivarius) channels are shown for each environment. Scale bar, 10 µm. **f** Quantification of aggregate size formed by Salivarius streptococci in communities grown in glucose-based medium versus mucin-based medium. Center lines indicate the medians, box limits indicate upper and lower quartiles, and whiskers indicate minimum and maximum values. Each point represents the average across an independent field of view. **g** Live confocal imaging of *S. salivarius* showing large aggregates in glucose-based medium and smaller group clusters in mucin-based medium. Scale bar, 10 µm. **h**, **i** Quantification of aggregate size (**h**) and distribution in z position (**i**) for *S. salivarius* grown in glucose-based or mucin-based medium. In (**h**), center lines indicate the medians, box limits indicate upper and lower quartiles, and whiskers indicate minimum and maximum values. In (**i**), each point represents the average and error bars represent the s.d. **j** *S. salivarius* growth on glucose- or mucin-based medium at 6 h. Each point represents an independent replicate.

In addition to nutritive function, mucins may modulate microbial communities by shaping spatial structure and thereby, impacting growth and competition. To probe effects on spatial distribution, we grew native oral communities in glucose-based medium with or without mucins, mucin glycans, or pooled monosaccharides, and biofilms were allowed to form on hydroxyapatite disks to mimic natural tooth enamel surfaces (Fig. 5a). Compared with medium alone, the biofilm fraction decreased when grown in the presence of mucins and mucin glycans, but not monosaccharides (Fig. 5b). The total biomass (planktonic and biofilm fractions) was similar or higher with mucins or glycans, revealing that the observed biofilm reduction is not due to limited total growth, but rather a shift from a biofilm to a planktonic state (Fig. 5b). Within these biofilm fractions, mucins and glycans also maintained more diverse communities and limited Salivarius outgrowth (Fig. 5c, d). We hypothesized that taxonomic diversity in the biofilm is related to the spatial distribution of bacteria.

To visualize changes in community structure, we applied multiplexed fluorescence in situ hybridization (FISH) to image biofilms formed by native salivary communities over 48 h. In the glucose-based medium, we found that Salivarius group streptococci formed large, thick aggregates (Fig. 5e, f, Supplementary Fig. 8). This is consistent with the Salivarius outgrowth observed in our 16S compositional analysis of planktonic and biofilm communities (Figs. 1i, 5c). In contrast, we found substantially less attached biomass and an absence of large Salivarius aggregates in the mucin-based medium (Fig. 5e, f, Supplementary Fig. 8).

To more closely monitor how mucin impacts the spatial organization of Salivarius streptococci, we performed live fluorescence imaging of *S. salivarius* (ATCC 13419). In the glucose-based medium, *S. salivarius* formed large aggregates orders of magnitude larger than those formed in the mucin-based medium (Fig. 5g, h), supporting the FISH observations of Salivarius streptococci within native communities. Moreover, *S. salivarius* in mucin was distributed above the surface, in contrast to the dense surface-attached aggregates observed in glucose-based medium (Fig. 5g, i). These differences in spatial distribution were likely not due to growth differences (Fig. 5j) nor physical constraints, as viscosity scales linearly with mucin concentration (over the range in concentration used for these experiments), a property characteristic of dilute polymer solutions, rather than entangled networks^35^ (Supplementary Fig. 9). Rather, a biochemical mechanism such as glycans serving as attachment sites or regulatory signals to shape growth behavior may trigger the observed spatial dispersion.

### Mucin glycans drive the assembly of communities featuring prolonged coexistence

**Fig. 6:**
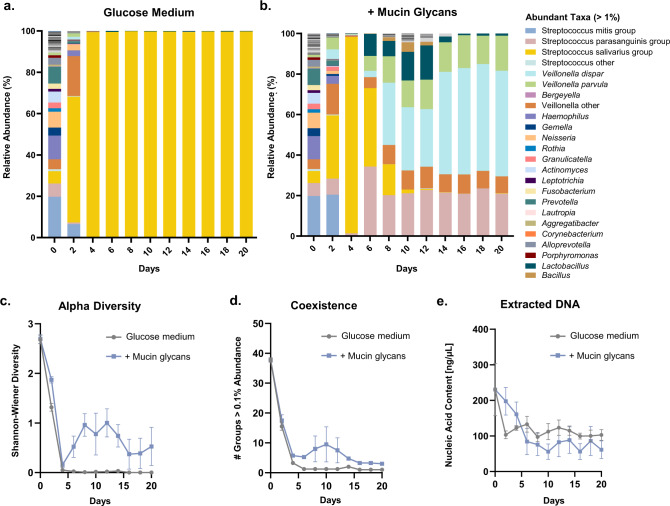
Mucin glycans promote prolonged microbial coexistence. **a**, **b** Relative abundance of taxa in microbial communities after serial passaging in glucose-based medium alone (**a**) or with mucin glycans (**b**). Bars show average relative abundances of four replicates including two inoculating communities. **c–e** Alpha diversity, (**c**) # of groups with abundance >0.1% (**d**), and extracted DNA content (**e**) from samples grown in each carbon environment of communities cultured with or without mucin glycans over 10 passages (20 days). In (**c–e**), each point represents the average and error bars indicate the s.e.m. (*n* = 4). All experiments were performed using inoculating communities 3 and 4, as depicted in Supplementary Fig. 1. The last three passages of one replicate community grown in glucose-based medium alone contained insufficient sequencing reads, and were excluded from analysis.

Each of the observed effects of mucin (e.g., diverse composition and metabolism, dispersed spatial distribution) can potentially promote coexistence within complex communities. However, batch culture experiments cannot capture prolonged interactions without the depletion of nutrients and host factors. For this, we used a serial passaging approach that facilitates the self-assembly of stable microbial communities^36^. Specifically, each community cultured in glucose medium with or without mucin glycans was propagated every 48 h for a total of 10 passages. Consistent with batch culturing, when salivary communities were passaged in glucose medium alone, Salivarius streptococci rapidly increased in abundance, outgrowing the culture after two passages and remaining dominant (at >99% abundance) for the entirety of the experiment (Fig. 6a).

In contrast, when passaged in glucose medium with mucin glycans, the oral communities assembled into stable states containing several dominant taxa within the *Streptococcus*, *Veillonella*, and *Lactobacillus* groups over 20 days (Fig. 6b). This corresponded to a marked increase in taxonomic diversity and coexistence, while total biomass remained comparable (Fig. 6c–e). The communities grown with mucin glycans assembled into two distinct stable states, depending on the starting inoculum (Supplementary Fig. 10). Specifically, inoculum 3 assembled into a state in which a low abundance of *Lactobacillus* coexisted with *Veillonella*, while inoculum 4 assembled into a state in which *Parasanguinis* streptococci coexisted with *Veillonella* (Supplementary Fig. 10). *Veillonella* cannot utilize glucose or mucin sugars as a carbon source and instead utilizes lactate^37^, a byproduct of *Streptococcus* and *Lactobacillus* metabolism^38^. Each of the four final communities shaped by mucin glycans contained lactic acid-producing and lactic acid-utilizing bacteria (Supplementary Fig. 10). Thus, while the precise interaction networks in the inoculating microbiota influenced the final composition, the stable states were functionally similar as we see sustained coexistence of metabolically cooperative bacterial groups in the presence of mucin glycans.

## Discussion

Mucus plays a critical role in maintaining microbial homeostasis across mucosal niches of the human body, but how mucus components impact community composition and structure has not been fully explored. To address this gap, we designed a highly tractable and physiologically-relevant culturing approach to assess how the host mediates microbial community assembly. We found that mucins and their glycans broadly modulate oral community composition, promoting diversity and retaining health-associated bacteria, while resisting microbial outgrowth in the presence of simple dietary sugars. Further, by integrating high-throughput sequencing data with metabolic phenotypes and visualization of spatial structure, we attained a multifaceted picture of how mucins and their glycans regulate our microbiota.

One mechanism by which mucins may facilitate microbial coexistence is providing complex nutritive substrates to enable niche partitioning^33^ and cooperative metabolism^9,11^. Here, we found growth of highly diverse communities when supplied isolated mucin glycans as a sole carbon source (Fig. 3). The inability of monosaccharide nutrient components to fully capture these effects demonstrates that the complex configuration of mucin glycans is essential. For example, structural complexity may require cooperation between microbes producing different linkage-specific glycosidases to effectively degrade and utilize mucin glycans. Indeed, many of the retained genera do not encode the putative mucin-targeting glycoside hydrolases needed for the complete glycan degradation (Fig. 3f, Supplementary Fig. 6), supporting the possibility of mucin glycans promoting community diversity through cooperative degradation and cross-feeding mechanisms. However, about half of the retained genera did not encode reported mucin glycan degradation machinery and are not known to utilize metabolic byproducts such as lactic acid. Furthermore, several studies have shown that bacteria capable of using mucin glycans prefer to consume dietary sugars when available^39,40^. Consequently, the nutritive role of mucin glycans may be masked in native saliva or gastrointestinal mucus, where dietary polysaccharides and fermentable sugars are often abundant.

In high-glucose conditions where nutritive functions may be partially masked, mucin glycans promoted the coexistence of different bacteria. Specifically, Mitis/Parasanguinis streptococci coexisted with other taxa when mucin glycans were supplied in both batch culture and serial passaging (Figs. 2h, 6b). If the retention of Mitis/Parasanguinis streptococci were due to their ability to utilize mucin glycans, then we would expect this group to be enriched for cultures in which glycans were the sole carbon source. Yet, Mitis/Parasanguinis streptococci were not retained when glycans were the sole carbon source, nor did the Mitis isolate benefit from glycans over 8 h (Fig. 3e, f, h). This indicates that mucin glycans support diverse communities through mechanisms beyond nutrition, potentially shaping cooperative and antagonistic microbial interactions through spatial organization or behavioral regulation. Multiple dual species models demonstrate mucins’ ability to attenuate competition^18,19,32^, which taken with the findings of this study, highlight how mucins and mucin glycans can facilitate microbial coexistence and support diverse communities.

Mucins’ regulation of community composition and structure may have important implications in oral cavity health. Salivary gland hypofunction commonly results in severe dental caries^41^, a condition also linked to reduced mucin production^42^. Here, we found that mucins and their glycans protected against two features associated with dental caries: decreased microbial diversity and a dysbiotic shift between Mitis and Salivarius streptococci^30^. Changes in oral streptococci biofilms have also been documented with dental caries progression^43^. Unlike in glucose medium, we found that in mucin, Salivarius streptococci do not form thick biofilms or aggregates when grown individually or within complex communities (Fig. 5). This suggests a role for mucins in managing oral community structure, possibly by presenting competitive binding sites, altering gene expression related to growth, and removing adhered bacteria (Supplementary Fig. 11). Future studies may elucidate the relevance of these different mechanisms. For instance, evidence of isolated glycans having similar effects on community spatial structure could underscore regulatory functions of mucin glycans.

Our observation that MUC5B, Muc5ac, and Muc2 each promote diversity is consistent with published work showing conserved function across mucin types (also isolated from different organisms)^16,18,44^. Intriguingly, the degree to which mucins and their glycans resisted dysbiosis and the specific microbes retained were influenced by the structural patterns in each glycan library (Fig. 2). Perhaps counterintuitively, we observed that Muc2 had the strongest effects on oral microbial community composition. One difference that we detected comparing the glycan preparations is that Muc2 displayed shorter, less extended glycans; 32% of Muc5ac glycans and 31% of MUC5B glycans contained more than five monosaccharides linked together, compared with less than 7% of Muc2 glycans (Supplementary Fig. 12). This characteristic difference may be due to microbial degradation in the porcine intestine or to differences in endogenous glycosyltransferase expression, but either way we speculate that these glycan structures are more easily accessible to the oral bacteria in our culture system. In the living host, mucin glycosylation profiles vary across body sites^45,46^. Thus, characteristic mucin glycosylation patterns may enable a mechanism for host selection of a healthy microbiota distinct to a given mucosal niche. This hints that the ability to tune glycosylation patterns represents a promising strategy for altering the composition and behavior of the microbiota to benefit host health.

## Methods

### Collection of inoculating microbial communities

We collected human saliva (5 mL) from healthy subjects through gentle aspiration with a custom vacuum pump. Subjects were asked to refrain from any food or drink 2 h before donating saliva. We collected the saliva samples after explaining the nature and possible consequences of the studies, obtaining written informed consent, and receiving approval from the institutional review board and Massachusetts Institute of Technology (MIT)’s Committee on the Use of Humans as Experimental Subjects under protocol #1312006096. For each inoculum, saliva samples from three donors were pooled. The composition of each inoculating community is depicted in Supplementary Fig. 1.

### Mucin purification

We purified native porcine gastric mucins (primarily Muc5ac, with small amounts of Muc2, Muc5B, and Muc6)^47,48^, porcine intestinal mucins (primarily Muc2)^18^, and human salivary mucins (primarily MUC5B, with small amounts of MUC7)^19^. We received approval for purification from porcine stomachs and intestines from the MIT’s Committee of Animal Care under protocol #E19-11-0722. Mucus was scraped from fresh pig stomachs and intestines and diluted (1 g scrapings to 5 mL) in 0.2 M sodium chloride containing antibacterial agents and protease inhibitors at the following concentrations: sodium azide (0.04 wt%), benzamidine HCl (5 mM), dibromoacetophenone (1 mM), phenylmethylsulfonyl fluoride (1 mM) and EDTA (5 mM, pH 7), and gently stirred overnight at 4 °C to solubilize the mucus. Insoluble material was removed by ultracentrifugation at 90,000 × *g* for 1 h at 4 °C (30,000 rpm, Beckman 45 Ti rotor with polycarbonate bottles). Submandibular saliva was collected in bulk (50 mL/sample) from human volunteers using a custom vacuum pump, pooled, and centrifuged, and antibacterial agents and protease inhibitors were added at the same concentrations as listed above. We purified mucins using size-exclusion chromatography on separate Sepharose CL-2B columns. Mucin fractions monitored by UV absorbance at 215 nm and verified by periodic acid-Schiff or phenol-sulfuric acid assays were then desalted, concentrated, and lyophilized for storage at −80 °C. Mass spectrometry is routinely used to monitor the composition of purified mucin extracts. For example, this type of analysis has shown that mucin purified from porcine gastric mucus is predominantly Muc5ac, with small quantities of Muc2, Muc5B, and Muc6, as well as histones, actin and albumin^47,48^.

### Isolation of mucin *O*-glycans

We applied non-reductive alkaline β-elimination ammonolysis, which preserves *O*-glycans in their native form, to dissociate non-reduced glycans from mucins^16,18,32,44^. Purified mucins were dissolved in 30–32% ammonium hydroxide solution saturated with 30% w/v ammonium carbonate and incubated at 60 °C for 40 h to release oligosaccharide glycosylamines and partially deglycosylated mucins. We removed volatile salts via repeated centrifugal evaporation then converted the resulting oligosaccharide glycosylamines to reducing oligosaccharide hemiacetals via treatment with 0.5 M boric acid at 37 °C for 1 h. Residual boric acid was removed via repeated centrifugal evaporation utilizing methanol. Next, free reducing glycans were separated by centrifugal filtration through 3 to 5 kDa molecular-weight cut-off membranes according to the manufacturer’s instructions (Amicon Ultracel). Glycans were further purified via solid-phase extraction through Hypercarb mini-columns (ThermoFisher), and residual solvents were removed through centrifugal evaporation. Glycans released from mucins were permethylated and analyzed by nanospray ionization tandem mass spectrometry (NSI-MS/MS) following direct infusion into a linear/orbital hybrid ion trap instrument (Orbitrap-LTQ Discovery, ThermoFisher) operated in positive ion mode as previously described^18,49^. Complete glycan structure profiles were deposited at GlycoPOST (#GPST000254).

### Partial acid hydrolysis of released mucin O-glycans

To isolate core glycan structures, we used partial acid hydrolysis^32^ in which different monosaccharides were hydrolyzed from intact glycans. To remove terminal sialic acid and fucose, we incubated glycans in 1 M trifluoroacetic acid at 80 °C for 4 h. Glycans were then neutralized with KOH, incubated at room temperature for 10 min to allow salts to precipitate, and centrifuged at 16,000 × *g* for 10 min to remove precipitant. Soluble glycans were purified through Hypercarb cartridges primed with four column volumes of 100% acetonitrile and flushed with four column volumes of water. We washed the Hypercarb cartridges with two column volumes of water and one column volume of 2% acetonitrile to remove salts and monosaccharides. Glycans were then eluted using two column volumes of 50% acetonitrile and dried via centrifugal evaporation.

### Batch culture

We grew native bacterial communities from healthy individuals in various controlled environments consisting of chemically-defined medium^27^ with glucose as the carbon source or without the addition of a carbon source (see Supplementary Methods for details on culture medium preparation, and Supplementary Table 1 for a complete list of medium components and concentrations). To prepare media containing mucins, lyophilized mucins were reconstituted by gentle shaking at 4 °C overnight in the medium described for each experiment, so that the polymers could fully rehydrate. Other additional components (glucose, glycans, hydrolyzed glycans, and monosaccharides) were mixed into the media immediately before use. We inoculated pooled saliva samples into 100 µL of culture medium with or without 0.25% (wt/vol) mucin, 0.1% (wt/vol) pooled mucin glycans isolated from the backbone, 0.1% (wt/vol) hydrolyzed glycans, or 0.2% (wt/vol) pooled monosaccharides present on mucin (including N-acetyl glucosamine, N-acetyl galactosamine, galactose, fucose, and sialic acid in equal concentrations; each monosaccharide was purchased from Sigma). For the no-carbon source experiments, mucins, isolated glycans, or monosaccharides were each supplied at 0.25% (w/v). Glucose was diluted from a 40% (w/v) stock solution in Milli-Q water; each of the other substrates were weighed out and added to solution. Cultures were incubated under anaerobic conditions for up to 48 h at 37 °C, and then collected via centrifugation for total DNA extraction. CFUs were counted on brain-heart infusion (BHI, BD) 1.5% agar plates.

### Serial passaging

To assess the membership of stabilized communities cultured with or without mucin glycans, we sequentially passaged healthy microbial communities. Aliquots (5 µL) of the starting saliva pool were inoculated into 100 µL of the chemically-defined glucose medium alone or supplemented with a pool of isolated mucin glycans. Cultures were grown anaerobically for 48 h at 37 °C. We homogenized each culture by pipetting up and down 10 times before passaging. Passaging was performed by taking 2 µL from each culture as inocula for 100 µL of fresh medium. We repeated this process every 48 h for a total of 10 passages. Samples were collected after each passage, flash frozen, and stored at −80 °C prior to DNA extraction and sequencing.

### Community membership analysis

We isolated total genomic DNA from bacterial samples at multiple time points from 0 to 48 h with an initial lysozyme treatment (ThermoFisher) and bead beating step with lysing matrix B (MP Biomedicals), followed by extraction with the MasterPure DNA Purification Kit (EPICENTRE) in accordance with the manufacturer’s instructions. The V4 region of the 16S rRNA gene was PCR amplified, and the resulting amplicons were cleaned, quantified, and sequenced on the Illumina MiSeq platform by the MIT BioMicro Center^50^. Raw sequences (300-bp paired-end reads) were processed, and taxonomic assignments were determined using QIIME2 (Quantitative Insights Into Microbial Ecology)^51^ and the Human Oral Microbe Database^52^. QIIME2 was also used to quantify diversity metrics. Prior to calculating FCs, a pseudocount of 0.001% (corresponding to <1 read) was added to each taxon to ensure the generation of real numbers. PCoA was performed using the QIIME2 pcoa plugin^53,54^ and weighted UniFrac distance metric, and visualized with Emperor^55,56^. Hierarchical clustering was performed and visualized using Morpheus (https://software.broadinstitute.org/morpheus). Unless otherwise noted, communities were clustered based on the relative abundance of each genera with >0.1% average abundance in at least one condition using the 1-Pearson distance metric and average linkage.

### Validation and growth of *Streptococcus* isolates

We cultured isolates from native communities identified as *S. salivarius* or *S. oralis/mitis/infantis* by sequencing of the ribosomal intergenic spacer region. To isolate these streptococci, oral communities from saliva were grown in medium with or without mucin glycans, homogenized by pipetting up and down, and then 1 µL of medium was used to isolate individual bacteria on Brucella blood agar plates with hemin, and vitamin K (VWR). After growth for 48 h in an anaerobic box at 37 °C, single colonies were picked and re-streaked onto Mitis-Salivarius agar (VWR), a selective medium that facilitates the growth and phenotypic identification of streptococci. After growth for 48 h in an anaerobic box at 37 °C, single colonies were picked and cultured in liquid medium. Once turbid, bacterial DNA was extracted, purified, and quantified by nanodrop. The intergenic spacer region between the large and the small subunit of ribosomal sequences (RIS) was then PCR amplified using the Phusion high fidelity polymerase Master Mix (NEB), purified template DNA, and the following primers: Forward (5′-TGCGGCTGGATCCCCTCCTT-3′) and Reverse (5′-CCGGGTTTCCCCATTCGG-3′)^57^. To amplify the gene product, the following cycling parameters were used: Initial denaturation, 98 °C for 3 m. 35 cycles: Denaturation, 98 °C for 10 s; Annealing, 56 °C for 30 s; Extension, 72 °C for 45 s. Final extension, 72 °C for 10 m. In total, 7 isolates were successfully obtained and sequenced. Alignment with BLAST identified 4 isolates as *Streptococcus salivarius*, and 3 isolates as *Streptococcus oralis*. Sequences aligning to the *Streptococcus oralis* RIS region also aligned at >99% identity to the *Streptococcus mitis* and *Streptococcus infantis* RIS regions. Isolates belonging to the Mitis and Salivarius group streptococci were grown in chemically-defined medium containing either glucose, mucin, isolated mucin glycans, or monosaccharides as the sole carbon source, at 37 °C under anaerobic conditions. For a single, representative strain of Mitis and Salivarius group streptococci, we measured CFU/mL on BHI 1.5% agar plates at 0, 8, and 24 h.

### Quantification of putative mucin-targeting glycoside hydrolases (GH)

For each representative bacterial strain corresponding to a dominant genus in the inoculum, a GH profile was obtained from the carbohydrate-active enzyme (CAZy) database^58^. The total number of GHs targeting linkages present in mucin were enumerated. GHs are classified in families based on amino acid similarities, which reflects the structural features of these enzymes in addition to substrate specificity. The specific GH families of interest were GH2 [β-gal], 18 [β-glcNAc], 20 [β-glcNAc, lacNAc, β-SO3-glcNAc], 27 [α- galNAc], 29 [α-fuc], 31 [α-galNAc], 33 [neu5ac], 35 [β-gal], 36 [α-galNAc], 42 [β-gal], 95 [α-fuc], 98 [β-gal], 101 [α-galNAc], 109 [α-galNAc], 112 [lacNAc], and 151 [α-fuc], where the target linkages are indicated in brackets.

### Metabolic profiling with Biolog EcoPlates

We grew native bacterial communities from human saliva in chemically-defined medium with the addition of either glucose, Muc5ac, isolated Muc5ac glycans, or monosaccharides for 24 h at 37 °C under anaerobic conditions. We diluted cultures 1:10 and inoculated onto Biolog EcoPlates (90 µL per well, three replicates per carbon source). We read the absorbance at 590 and 750 nm every 24 h for 96 h. To avoid artificial differences caused by varying inoculating density, the absorbance values were corrected by 750-nm readings and normalized by a blank well containing inoculating communities but no-carbon source.

### Biofilm biomass analysis

To monitor community biofilm formation, we grew native oral communities in glucose-based medium with or without Muc5ac, Muc5ac glycans, or pooled monosaccharides comprising mucin glycans, allowing biofilms to form on hydroxyapatite disks 5 mm in diameter and 2 mm thick (Clarkson Chromatography Products) used to mimic tooth enamel. At various time points, we removed the planktonic fraction, washed the remaining attached fraction with phosphate-buffered saline to remove loosely adherent cells, and detached cells from the disks by vortexing and sonification. We quantified total bacterial load in the culture and bacterial load in the biofilm based on the total DNA content^59,60^ via nanodrop methods.

### Community biofilm fixation for FISH

We grew native bacterial communities from human saliva in chemically-defined medium with either glucose or MUC5B for 48 h at 37 °C under static, anaerobic conditions in glass-bottom 96-well plates. Samples were fixed following published methods^25^, using 2% (wt/vol) paraformaldehyde in 10 mM Tris (pH 7.5) for 1.5 h on ice. We gently washed samples 1 × 10 mM Tris (pH 7.5) for 15 min, allowed the sample to settle by gravity, and removed the supernatant. We stored samples in 50% (vol/vol) ethanol and air-dried them immediately before FISH.

### FISH, image acquisition, and analysis

We purchased fluorophore-labeled oligonucleotides from www.biomers.net. We applied standard FISH protocols^25^ on biofilm samples formed by native oral bacterial communities on glass-bottom 96-well plates. We covered samples with a hybridization solution [900 mM NaCl, 20 mM Tris, pH 7.5, 0.01% SDS, 20% (vol/vol) formamide, each probe at a final concentration of 2 nM] and incubated for 2.5 h at 46 °C. We washed samples with a wash buffer (215 mM NaCl, 20 mM Tris pH 7.5, 5 mM EDTA), incubating for 15 min at 48 °C. The samples were then rinsed with ice cold water followed by 100% ethanol, air-dried, coated in ProLong Gold Antifade Solution (ThermoFisher), and cured overnight in the dark at 4 °C. Supplementary Fig. 8 shows additional fields of view for FISH staining of native oral communities, validation of probe target specificity using individual *Streptococcus* strains, as well as a table reporting each probe sequence, target organism, fluorophore, and reference^26,61,62^. Images were acquired using a confocal laser scanning microscope (Zeiss LSM 800) equipped with a 63×/1.4 NA oil immersion objective. Each field of view was imaged using an excitation wavelength of 405 nm for the DY-415-labeled universal bacterial probe, 488 nm for DY-490-labeled probe targeting *S. mitis/oralis/infantis*, 563 nm for Atto-550-labeled probe targeting *S. salivarius/vestibularis* and 635 nm for Atto-633-labeled probe targeting *Streptococcus*. We analyzed images with Zeiss ZEN 2.1 imaging software (RRID:SCR_013672) and performed biofilm quantification and 3D reconstruction of bacterial biomass using IMARIS 9.9.1 (RRID:SCR_007370).

### Imaging *S. salivarius* structure

We diluted overnight cultures of *S. salivarius* (derived from ATCC® 13419) 100-fold in glucose-based or mucin-based medium in glass-bottom 96-well plates and incubated for 6 h at 37 °C under static, anaerobic conditions. We stained biofilms for 30 min with Syto9 (ThermoFisher) prior to imaging. Images were acquired using a confocal laser scanning microscope (Zeiss LSM 800) equipped with a 63×/1.4 NA oil immersion objective. We used an excitation wavelength of 488 nm for Syto9 and a step size of 0.5 μm. We analyzed images with Zeiss ZEN 2.1 imaging software (RRID:SCR_013672) and performed biofilm quantification and 3D reconstruction of bacterial biomass using IMARIS 7.7.2 (RRID:SCR_007370).

### Mucin gel microstructural analysis

We prepared mucin samples at concentrations from 0 to 0.5% (wt/vol) MUC5B in 20 mM 4-(2-hydroxyethyl)-1-piperazineethanesulfonic acid (HEPES) and 20 mM NaCl at pH 7. We added fluorescent, negatively charged (carboxylated) microspheres 1 µm in diameter (Magsphere Inc.) at an overall dilution ratio of 1:12,000, vortexed samples, then pipetted into borosilicate square capillaries 0.9 mm × 0.9 mm × 15 mm (Vitrocom). Capillaries were sealed on both ends using a 1:1:1 mixture of petroleum jelly, lanolin, and paraffin, and then mounted onto microscope slides for imaging.

We performed single particle tracking following published methods^63^. Briefly, we acquired images using a Zeiss Axio Observer D.1 inverted microscope with a Zeiss LD Plan-Neofluar 20x/0.4 Corr Ph2 objective lens (Carl Zeiss Microscopy GmbH) and a Hamamatsu Flash 4.0 C11440–22CU camera (Hamamatsu Photonics). Imaging was performed at 30.3 frames per second for 10 s at room temperature. At least 150 particles were imaged for each sample from an average of 10 different locations within the capillaries. We used a publicly available MATLAB code^64,65^ to identify particles in each image frame and track individual trajectories. We calculated the ensemble averaged MSD overall particles in each sample and extracted the translational diffusion constant (D). From these diffusion constants, we calculated the viscosity (µ) of the mucin environments using the Stokes–Einstein relation. Using these viscosities and Stokes’ law, we calculated and approximate settling velocity for a bacterial cell through each mucin environments, and the corresponding settling time for a distance of 100 µm (the highest biomass visualized in Fig. 5g). Equations and calculations are shown in Supplementary Fig. 9.

### Statistical analysis

Unless otherwise noted, we assessed significant changes in diversity relative to medium alone using repeated-measures one-way ANOVA with Dunnett’s multiple comparison test. Significant changes in taxa abundance were identified with one-way, two-sided *t*-tests. We adjusted the significance threshold with Bonferroni’s correction for multiple comparisons.

## Supplementary information

Supplementary Material

## Data Availability

Raw 16S sequencing data are available in the DDBJ Sequence Read Archive (DRA) under BioProject accession number PRJNA892622. MS data of the mucin glycan profiles are deposited at GlycoPOST under accession number GPST000254. All other data are available from the corresponding author upon reasonable request.
