# Supplementary material for: Mucin glycans drive oral microbial community composition and function
Source: NPJ Biofilms Microbiomes. 2023 Mar 23;9:11. doi: 10.1038/s41522-023-00378-4 (PMC10036478; doi:10.1038/s41522-023-00378-4)
Supplement: Supplementary file 1 — Supplementary Material [file 41522_2023_378_MOESM1_ESM.pdf]

Supplementary Methods

Preparation of culture medium. Salt stock solutions were prepared at 50x concentration by weighing out the salts, stirring and dissolving fully in MilliQ water, then adding MilliQ water to the final volume. The following salt stock solutions were prepared separately: phosphate ([$K_2HPO_4 \cdot 3H_2O$], [KH_2PO_4]), chloride ([NH_4Cl], [$MgCl_2$], [$MnCl_2 \cdot 4H_2O$], [$CaCl_2$]), pyruvate ([$C_3H_3NaO_3$]), carbonate ([Na_2CO_3]), iron ([$FeSO_4 \cdot 7H_2O$]), and urea ([CH_4N_2O]). Phosphate, chloride, and pyruvate stock solutions were autoclaved for 15 min; carbonate, iron, and urea stock solutions were sterilized through a 0.2 µm syringe filter. 20x amino acid (Glu, Cys, Leu, Lys, Arg, Pro, Gly), 20x L-cysteine, and 100x base (adenine) stock solutions were prepared by weighing out each component into MilliQ water with continuous stirring, and sterilizing through a 0.2 µm syringe filter. All stock solutions were stored at 4 °C for up to 1 month. A commercial mix of vitamins at 10x concentration was purchased from ATCC (MD-VS). Stock solutions were combined to prepare the complete medium, which was stored at 4 °C for up to 1 week. Glucose was diluted from a 40% (w/v) stock solution in Milli-Q water and added to the medium immediately before use. A complete list of components and their concentrations is provided in **Supplementary Table 1**.

Supplementary Figures

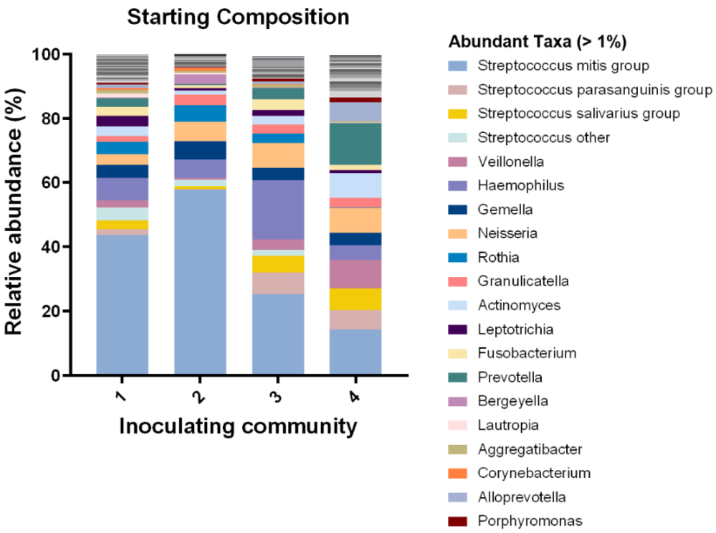

Supplementary Figure 1. Starting composition of each inoculating community. Relative abundance from 16S rRNA sequencing of inoculating oral microbial communities. For each community, saliva samples from three donors were pooled. Each stacked bar represents the average relative abundances across two replicates.

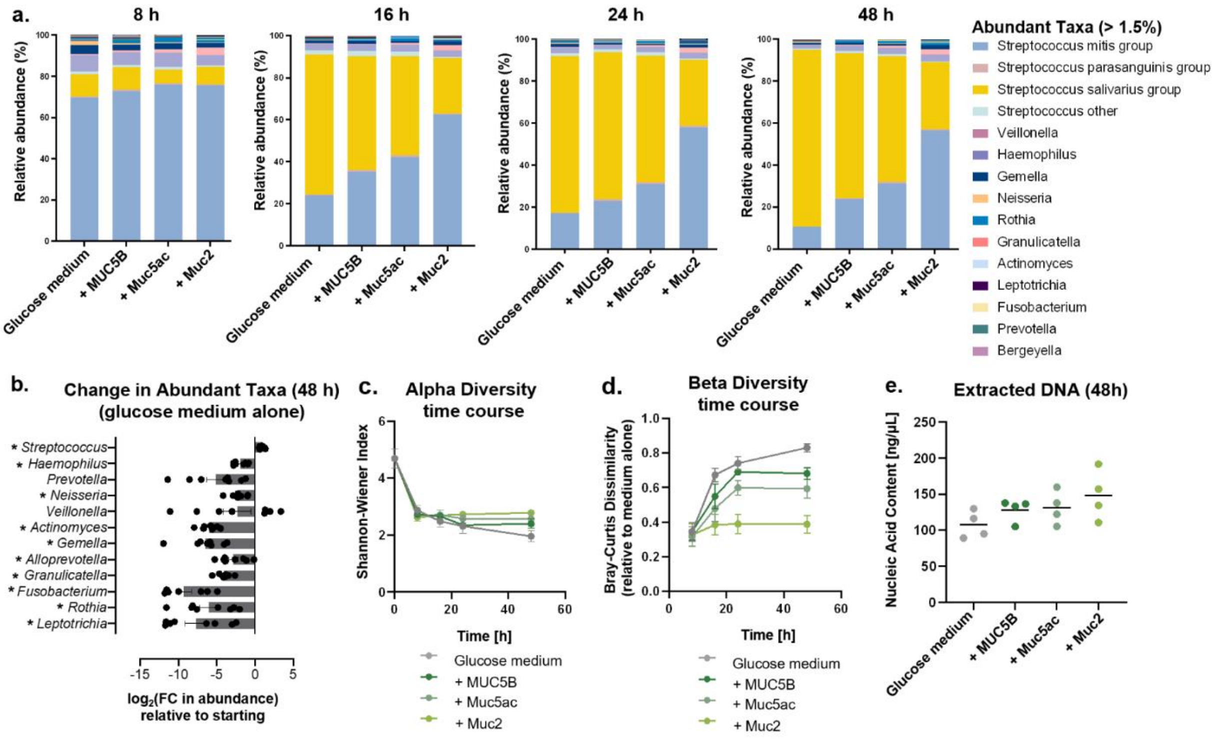

Supplementary Figure 2. Mucins maintain more diverse oral microbial communities over 48 h. (a) Relative abundance from 16S rRNA sequencing of oral microbial communities grown in medium with or without mucins over time. Planktonic fractions were analyzed at the time points: 8, 16, 24, and 48 h. Each stacked bar represents the average relative abundances including replicates from inoculating communities 1 and 2 (n=4). **(b)** Change in abundance of dominant genera (>1% in starting inocula) after growth in 48 h in glucose medium without mucins. Bar length represents the mean change in abundance, each point represents the change for an independent replicate, and error bars indicate standard error of the mean (s.e.m.). Significant changes in taxa abundance were identified with one-way, two-sided t-tests. Significance threshold was adjusted with Bonferroni correction for multiple comparisons. * p<0.0042. **(c,d)** Alpha **(c)** and Beta **(d)** diversity of microbial communities cultured in medium with or without mucins at time points: 8, 16, 24, and 48 h. In **(c,d)**, each point represents the average across three or four replicates†, and error bars represent the s.e.m. **(e)** Total extracted DNA quantity in glucose medium with or without mucins. In **(e)** each point represents an independent replicate (n=4).

†Muc5ac and Muc2 at 8 h include three replicates; all other samples include four replicates

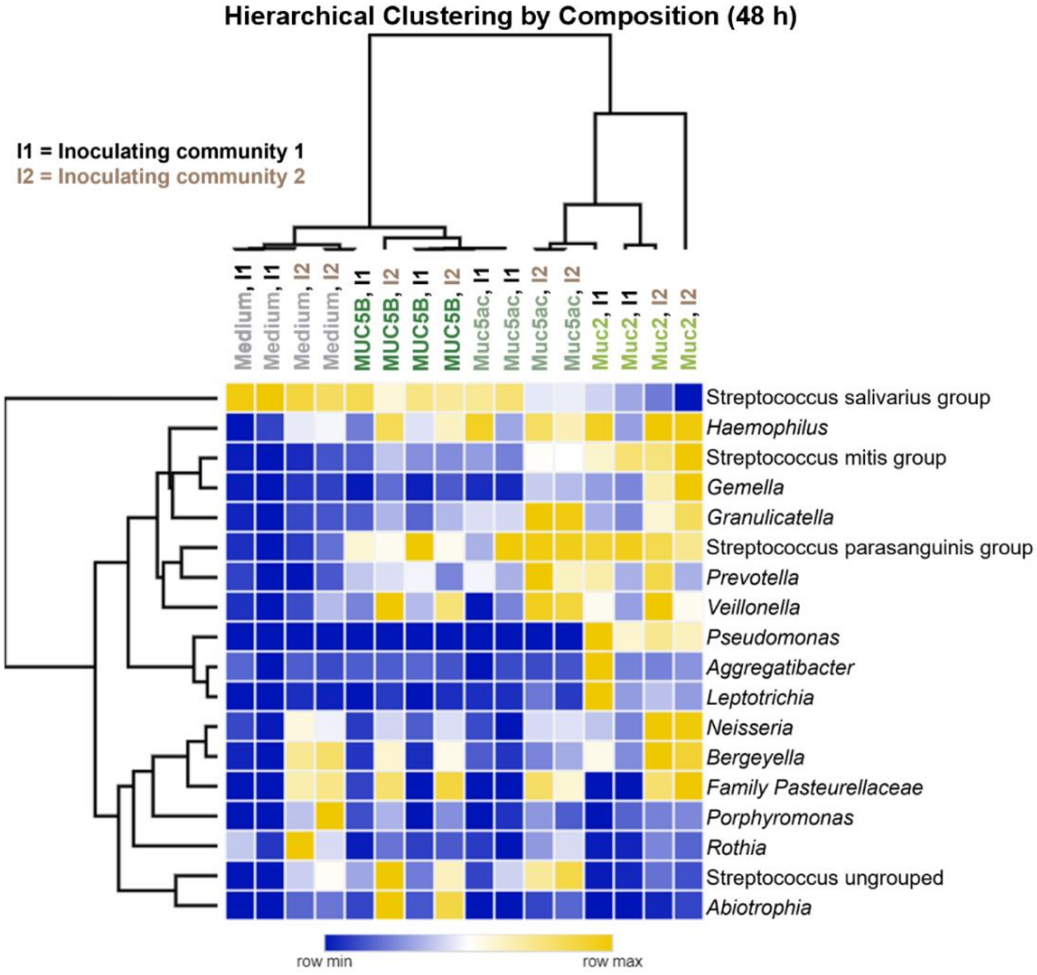

Supplementary Figure 3. Glycan patterns from different mucosal tissues correlate with microbial composition. Microbial communities cluster according to the culture medium. The dendrogram across columns represents average linkage clustering (1-Pearson distance metric) of communities based on microbial composition (48 h). The dendrogram across rows represents average linkage clustering (1-Pearson distance metric) of taxa based on abundance profiles across samples. The heatmap shows the relative abundance of microbial taxa at >0.1% abundance (48 h). Each column represents an independent replicate.

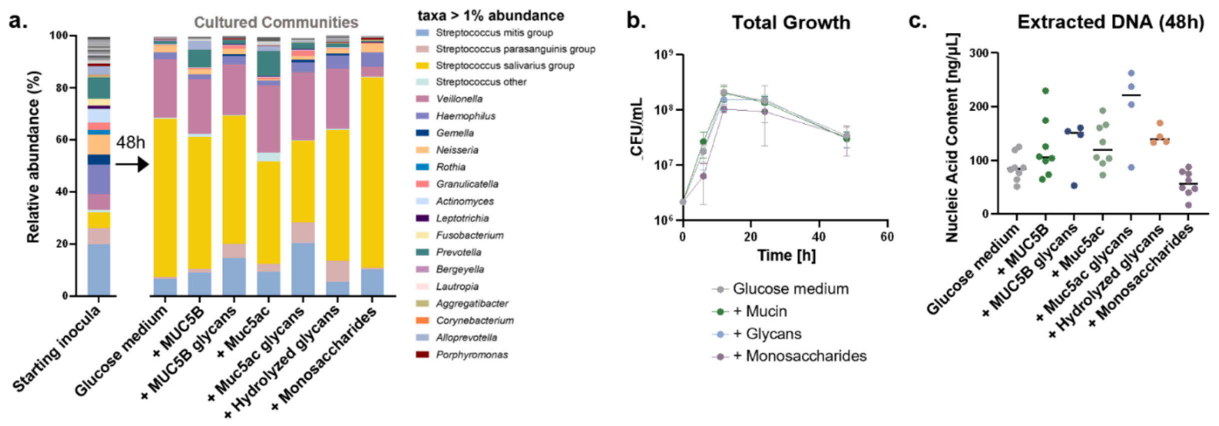

Supplementary Figure 4. Mucin glycan pools shape community composition without impacting overall growth. (a) Relative abundance of microbial communities cultured with mucins or mucin glycans at 48 h. Bars show average relative abundances of four replicates including two inoculating communities. **(b)** Total growth of the microbial community in each culture medium. Each point represents the average CFU/mL (n=3), and error bars indicate the standard deviation (s.d.). **(c)** Total extracted DNA quantity for each culture environment. Analysis for glucose medium, MUC5B, Muc5ac, and monosaccharides includes duplicates for each inoculating community 1–4 (n=8). Analysis for MUC5B glycans, Muc5ac glycans, and hydrolyzed glycans includes inoculating communities 3 and 4 (n=4). Each point represents an independent replicate. In **(c)**, data for medium alone, MUC5B, and Muc5ac with inoculating communities 1 and 2 are duplicated from **Supplementary Figure 2** for comparison.

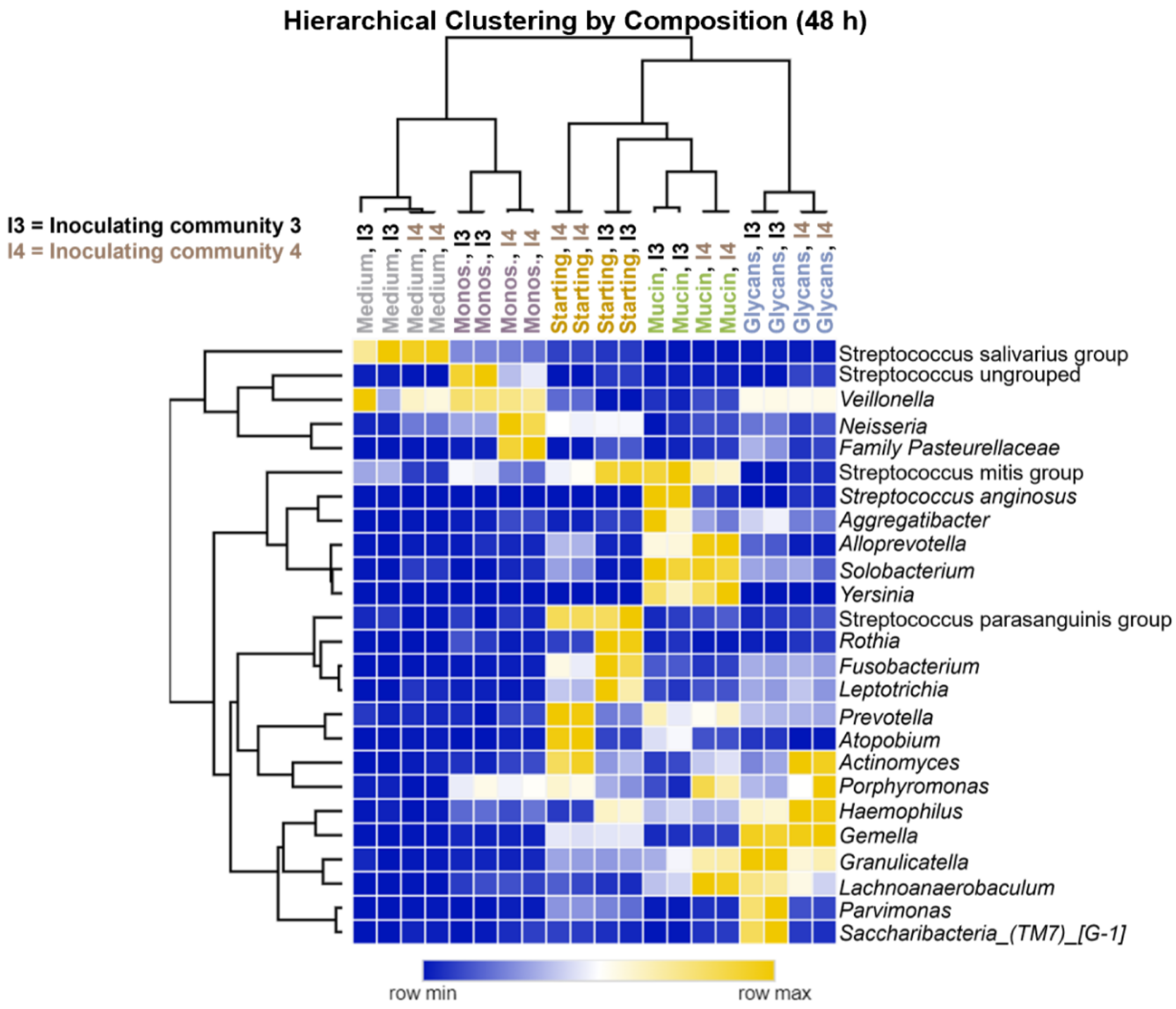

Supplementary Figure 5. Microbial communities clustered according to sole carbon source (48 h). The dendrogram across columns represents average linkage clustering (1-Pearson distance metric) of communities based on microbial composition. The dendrogram across rows represents average linkage clustering (1-Pearson distance metric) of taxa based on abundance profiles across samples. The heatmap shows the relative abundance of microbial taxa at >0.1% abundance (48 h). Each column represents an independent replicate.

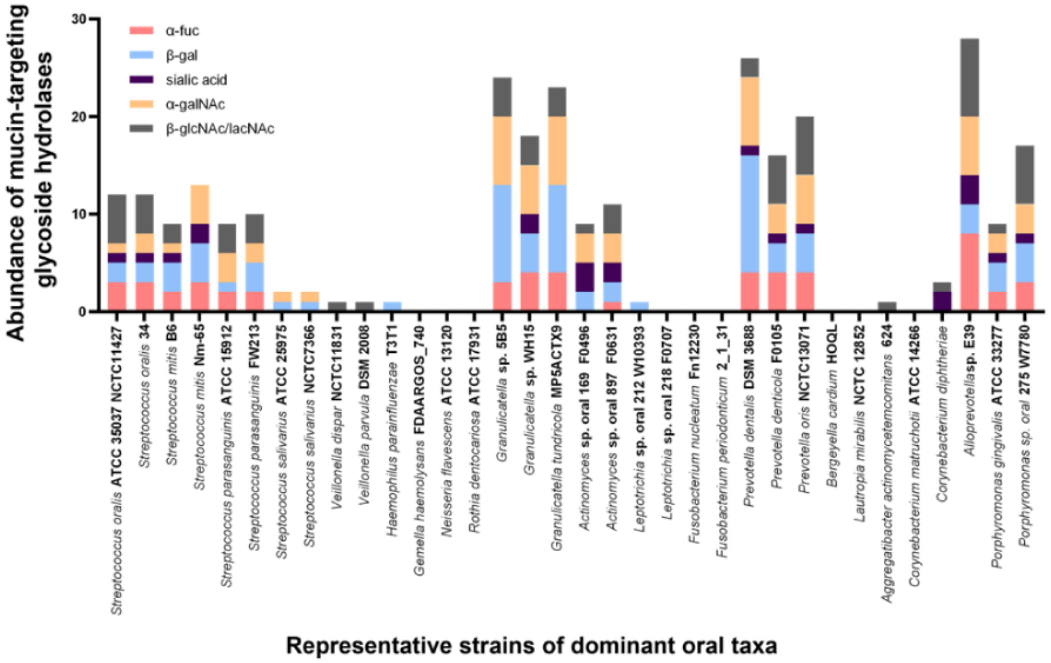

Supplementary Figure 6. Abundance of putative mucin-targeting glycoside hydrolases (GHs) in representative oral bacteria. For each representative bacterial strain, a GH profile was obtained from the carbohydrate-active enzyme (CAZy) database[1]. Mucin-targeting GH families include: 2 [β-gal], 18 [β-glcNAc], 20 [β-glcNAc, lacNAc, β-SO3-glcNAc], 27 [α- galNAc], 29 [α-fuc], 31 [α-galNAc], 33 [neu5ac], 35 [β-gal], 36 [α-galNAc], 42 [β-gal], 95 [α-fuc], 98 [β-gal], 101 [α-galNAc], 109 [α-galNAc], 112 [lacNAc], and 151 [α-fuc], where the target linkages are indicated in brackets. Each bar represents the total number of enzymes identified targeting a linkage type.

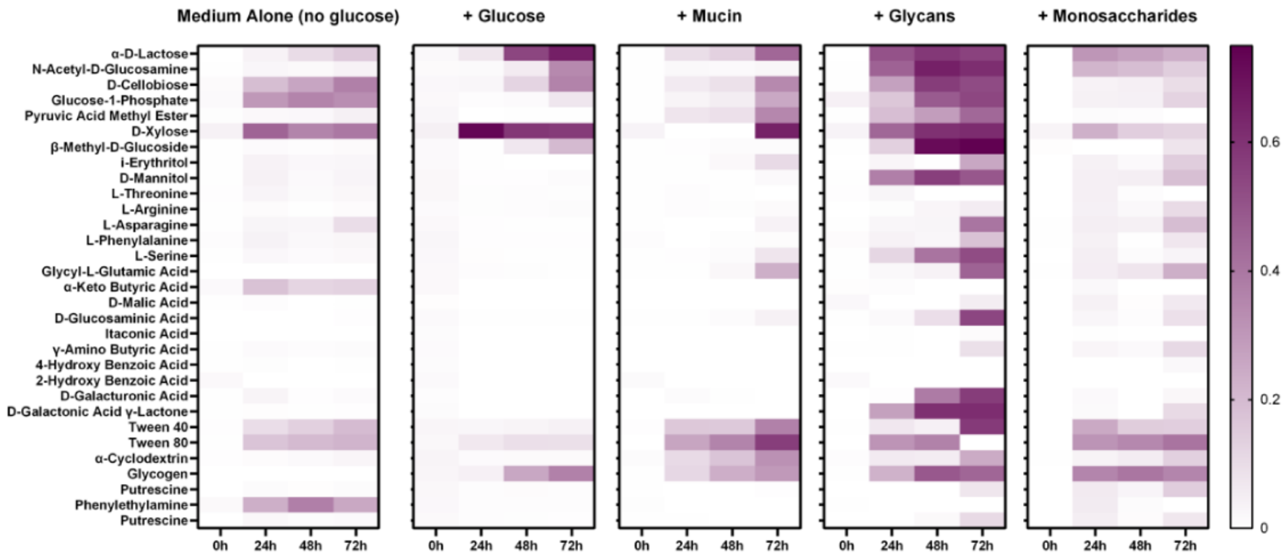

Supplementary Figure 7. Carbon source utilization profiles over time of communities shaped by different culture environments. Heatmap shows normalized absorbance values of carbon utilization measured using Biolog EcoPlates for each microbial community from 0–72 h. Each sample represents the average of three replicates.

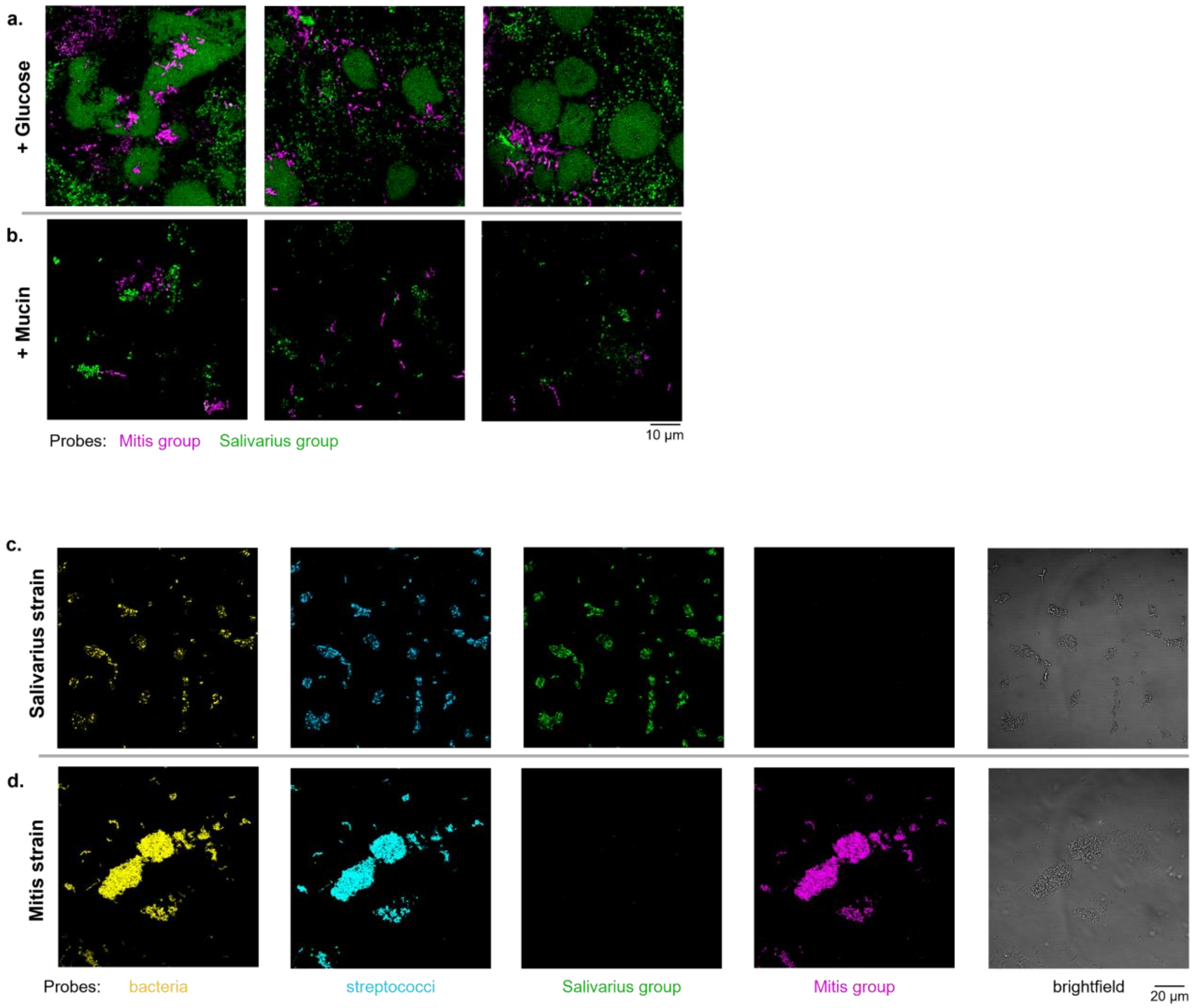

e.

Probe name	Sequence (5' to 3')	Target	Fluorophore	Reference
Smit651	CCCCTCTTGCACTCAA	*S. mitis/oralis/infantis*	DY-490	(Wilbert et al. 2020)[2]
Ssal372	AGGGTTGCCCCCATT	*S. salivarius/vestibularis**	Atto 550	(Wilbert et al. 2020)[2]
Str405	TAGCCGTCCCTTTCTGGT	*Streptococcus*	Atto 633	(Paster et al. 1998)[3]
Eub338	GCTGCCTCCCGTAGGAGT	Bacteria	DY-415	(Amann et al. 1990)[4]

*also binds to taxa outside of *Streptococcus* including *Leptotrichia*, *Neisseria*, *Haemophilus*, and *Aggregatibacter*

Supplementary Figure 8. Additional material related to FISH experiments. (a,b) Additional fields of view for FISH staining of native oral communities grown in **(a)** glucose-based medium or **(b)** mucin-based medium. Merged green (Salivarius group) and magenta (Mitis group) channels shown. Scale bar, 10 µm. **(c,d)** Validation of FISH probes using individual *Streptococcus* strains belonging to the **(c)** Salivarius group and **(d)** Mitis group. Scale bar, 20 µm **(e)** FISH probes used in this study.

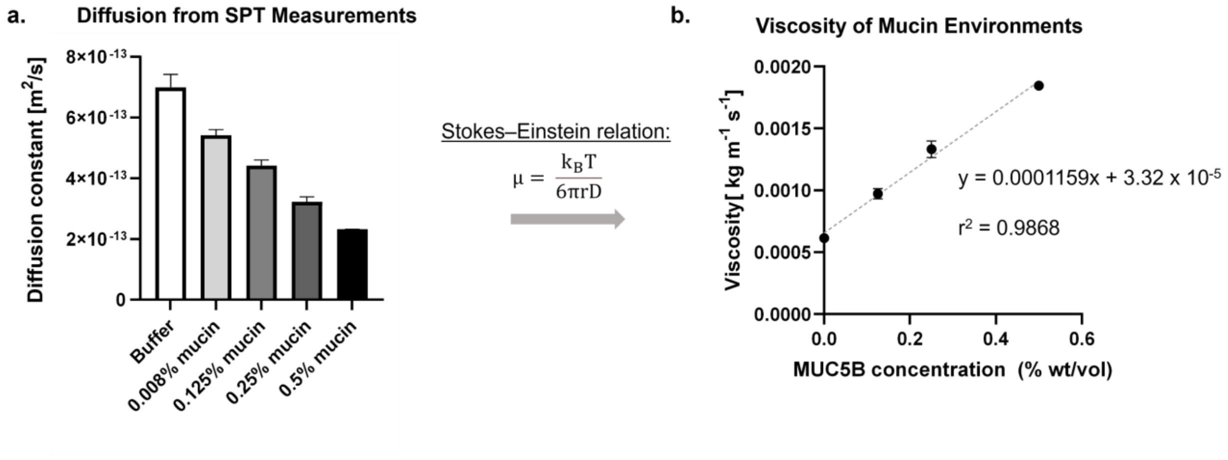

c.

Stokes' law to determine settling velocity: $v = \frac{2}{9}\frac{(\rho_p - \rho_f)}{\mu} g r^2$

Supplementary Figure 9. Microstructural analysis of mucin environments. (a) Diffusion constants of polystyrene beads (1 µm) for increasing concentrations of MUC5B, based on single particle tracking analysis. Each bar represents the average of two replicates and error bars represent the s.d. **(b)** Viscosity scales linearly with mucin concentration over the range of concentrations used in oral community experiments. Each point represents the average of two replicates, and error bars indicate the s.d. **(c)** Settling velocity was estimated using Stokes' law, and the corresponding time for a bacterial cell to settle over a distance of 100 µm was calculated to be approximately 10 min. This equation describes a small spherical particle moving through fluid with a small Reynolds number.

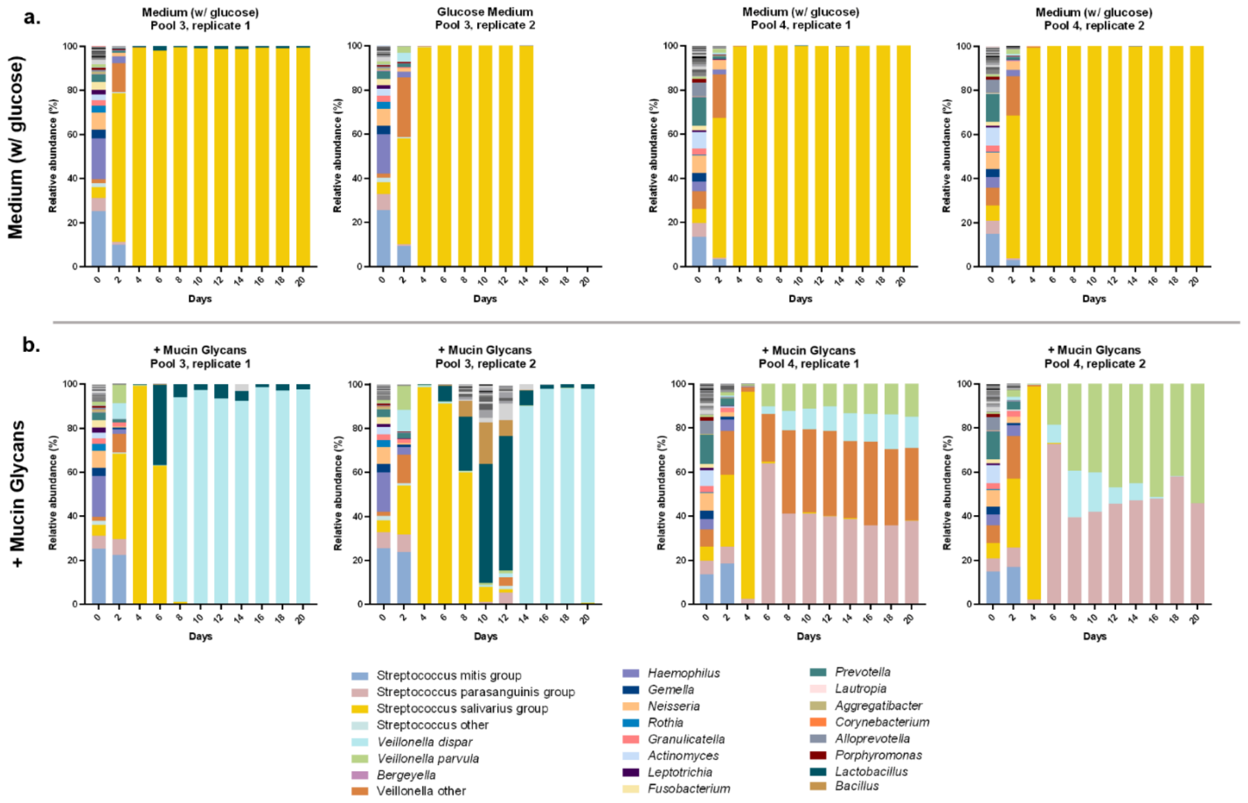

Supplementary Figure 10. Composition analysis of microbial communities over 10 serial passages in glucose medium alone (a) or with mucin glycans (b). Each graph represents an independent serial passaging experiment with inoculating community 3 or 4, as indicated. In **(a)**, the last three passages of the community “Pool 3, replicate 2” contained insufficient sequencing reads, and were excluded from analysis.

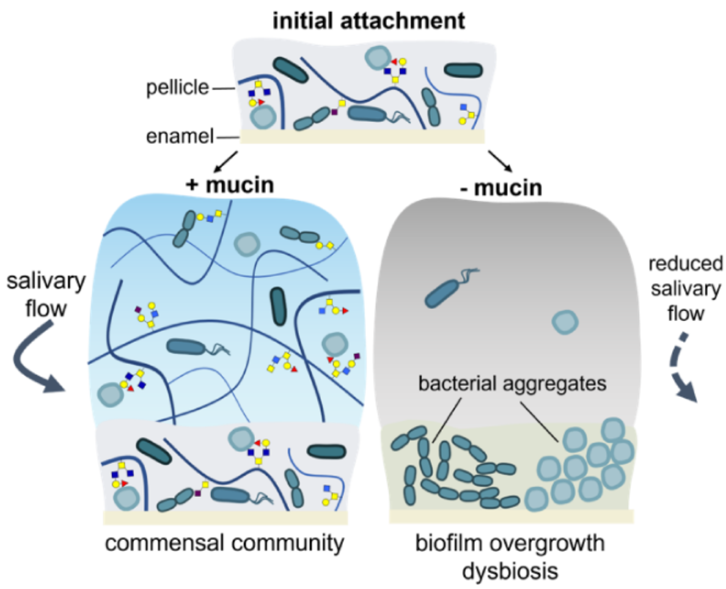

Supplementary Figure 11. Proposed model depicting mucins' roles in managing oral community structure by limiting surface attachment and aggregation. This schematic contains components (mucin polymers, bacteria) that are adapted with permission[5], originally published in *The FEBS Journal*.

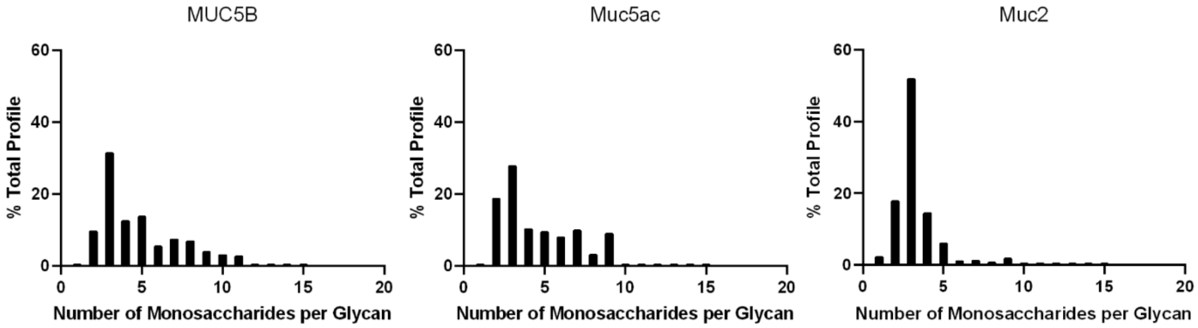

Supplementary Figure 12. Histograms showing number of monosaccharides linked together per glycan in MUC5B, Muc5ac, and Muc2 glycan preparations.

Supplementary Tables

Component	Concentration	Unit
Potassium phosphate dibasic trihydrate [$K_2HPO_4 \cdot 3H_2O$]	11.9	mM
Potassium phosphate monobasic [KH_2PO_4]	21.5	mM
Ammonium chloride [NH_4Cl]	10	mM
Magnesium chloride [$MgCl_2$]	5.9	mM
Magnesium chloride tetrahydrate [$MnCl_2 \cdot 4H_2O$]	0.1	mM
Calcium dichloride [$CaCl_2$]	1	mM
Sodium pyruvate [$C_3H_3NaO_3$]	5.5	mM
Iron(II) sulfate heptahydrate [$FeSO_4 \cdot 7H_2O$]	0.1	mM
Urea [CH_4N_2O]	5	mM
Sodium carbonate [Na_2CO_3]	1	g/L
Glutamic acid	2.6	g/L
Cysteine	0.4	g/L
Leucine	0.15	g/L
Lysine	0.5	g/L
Arginine	0.21	g/L
Proline	0.006	g/L
Glycine	0.004	g/L
Adenine	0.009	g/L
Folic acid	0.002	g/L
Pyridoxine hydrochloride	0.01	g/L
Riboflavin	0.005	g/L
Biotin	0.002	g/L
Thiamine	0.005	g/L
Nicotonic acid	0.005	g/L
Calcium pantothenate	0.005	g/L
Vitamin B12	0.0001	g/L
p-Aminobenzoic acid	0.005	g/L
Tioctic acid	0.005	g/L
Monopotassium phosphate	0.9	g/L
L-cysteine	0.5	g/L
Glucose	5	g/L

Supplementary Table 1. Composition of chemically-defined growth medium for oral microbial communities.

		Forward		Reverse		
**Experiment(s)**	**Target Region**	**Name**	**Sequence (5' to 3')**	**Name**	**Sequence (5' to 3')**	**Reference**
16S sequencing	16S V4	U515F	GTGCCAGCMGCCGCGGTAA	E786R	GGACTACHVGGGTWTCTAAT	(Caporaso et al. 2010)[6]
Isolate identification	intergenic spacer (RIS)	S-D-Bact-1522-b-S-20	TGCGGCTGGATCCCCTCCTT	L-D-Bact-132-a-A-18	CCGGGTTTCCCCATTCGG	(Cardinale et al. 2004)[7]

Supplementary Table 2. Complete list of primers used in the study.

Supplementary References

1. Lombard, V., Golaconda Ramulu, H., Drula, E., Coutinho, P. M. & Henrissat, B. The carbohydrate-active enzymes database (CAZy) in 2013. *Nucleic Acids Res* **42**, D490–D495 (2014).

2. Wilbert, S. A., Mark Welch, J. L. & Borisy, G. G. Spatial Ecology of the Human Tongue Dorsum Microbiome. *Cell Rep* **30**, 4003-4015.e3 (2020).

3. Paster, B. J., Bartoszyk, I. M. & Dewhirst, F. E. Identification of oral streptococci using PCR-based, reverse-capture, checkerboard hybridization. *Methods in Cell Science 1998 20:1* **20**, 223–231 (1998).

4. Amann, R. I. *et al.* Combination of 16S rRNA-targeted oligonucleotide probes with flow cytometry for analyzing mixed microbial populations. *Appl Environ Microbiol* **56**, 1919–1925 (1990).

5. Wang, B. X., Wu, C. M. & Ribbeck, K. Home, sweet home: how mucus accommodates our microbiota. *FEBS J* febs.15504 (2020) doi:10.1111/febs.15504.

6. Caporaso, J. G. *et al.* Global patterns of 16S rRNA diversity at a depth of millions of sequences per sample. *Proc Natl Acad Sci U S A* **108**, 4516–4522 (2011).

7. Cardinale, M. *et al.* Comparison of different primer sets for use in automated ribosomal intergenic spacer analysis of complex bacterial communities. *Appl Environ Microbiol* **70**, 6147–6156 (2004).
